# Reproducibility of biophysical *in silico* neuron states and spikes from event-based partial histories

**DOI:** 10.1371/journal.pcbi.1011548

**Published:** 2023-10-12

**Authors:** Evan Cudone, Amelia M. Lower, Robert A. McDougal

**Affiliations:** 1 Program in Computational Biology and Bioinformatics, Yale University, New Haven, Connecticut, United States of America; 2 Yale College, Yale University, New Haven, Connecticut, United States of America; 3 Department of Biostatistics, Yale School of Public Health, New Haven, Connecticut, United States of America; 4 Section of Biomedical Informatics and Data Science, Yale School of Medicine, New Haven, Connecticut, United States of America; 5 Wu Tsai Institute, Yale University, New Haven, Connecticut, United States of America; Université Paris Descartes, Centre National de la Recherche Scientifique, FRANCE

## Abstract

Biophysically detailed simulations of neuronal activity often rely on solving large systems of differential equations; in some models, these systems have tens of thousands of states per cell. Numerically solving these equations is computationally intensive and requires making assumptions about the initial cell states. Additional realism from incorporating more biological detail is achieved at the cost of increasingly more states, more computational resources, and more modeling assumptions. We show that for both a point and morphologically-detailed cell model, the presence and timing of future action potentials is probabilistically well-characterized by the relative timings of a moderate number of recent events alone. Knowledge of initial conditions or full synaptic input history is not required. While model time constants, etc. impact the specifics, we demonstrate that for both individual spikes and sustained cellular activity, the uncertainty in spike response decreases as the number of known input events increases, to the point of approximate determinism. Further, we show cellular model states are reconstructable from ongoing synaptic events, despite unknown initial conditions. We propose that a strictly event-based modeling framework is capable of representing the complexity of cellular dynamics of the differential-equations models with significantly less per-cell state variables, thus offering a pathway toward utilizing modern data-driven modeling to scale up to larger network models while preserving individual cellular biophysics.

## Author summary

There are 86 billion neurons in the human brain, each of which is a complex cell that responds to synaptic stimuli of other neurons with electrical signals. Neuroscientists have uncovered many of the biophysical mechanisms underlying the neuron’s function and utilize them to create detailed simulations of neurons and neural networks. However, due to the complexity of the neuron, these simulations require extensive computational resources. In this paper, we present a new modeling framework that incorporates the discrete synaptic events of neurons with the detailed mechanistic modeling of their biophysics, using a strictly event-based protocol. We investigate the extent to which this new event-based framework behaves like the existing conductance-based modeling approach by measuring how much its future responses vary provided different numbers of past input stimuli. This framework is an entry point for integrating machine learning methods to assist in reducing the computational complexity of detailed neuron modeling. The goal of this work is to enable researchers to better integrate mechanistic understanding of cell biology into broader multiscale studies.

## Introduction

Computational neuroscience employs a variety of modeling strategies to simulate neurons and networks. One such strategy, conductance-based neuron modeling, uses systems of differential equations with terms that represent the electrical and biophysical properties of excitable neurons. Such models are fundamental building-blocks for bottom-up design approaches in the modeling of the nervous system. Algorithmic advances [1–4], parallelized computing [5–7], and modern computing hardware [8–11] have advanced our ability to expeditiously run conductance-based models, but scaling such simulations to efficiently engage in the phenomenological study of the brain remains a challenge. Network simulations of conductance-based neuron models are possible given sufficient compute resources; e.g. Traub et al. 2005 (3.5k neurons) [12], Hill & Tononi 2005 (65k neurons) [13], Migliore et al. 2014 (13k-120k neurons) [14], Potjans & Diesmann 2014 (80k neurons) [15], Markram et al. 2015 (31k neurons) [16], Yang et al. 2019 (1m neurons on specialized hardware) [17], and Billeh et al. 2020 (230k neurons) [18]. However, simulating the collective 86 billion neurons of the human brain with morphological and biophysical detail requires orders of magnitude more processing power than what is currently feasible.

One alternative to simulating large systems of differential equations is to use event-based modeling. Event-based neuron models respond to input events (synaptic stimuli events) with output events (resulting action potentials). Modeling synaptic communication using only events can accelerate network simulation by decoupling the simulation of individual cells in a network, making it possible to simulate large neuronal networks in parallel [19]. Further efficiency at the cost of biophysical interpretability may be gained by replacing the calculus of the conductance-based models with a simplified rule, such as the analytical and algebraic solutions to the Integrate and Fire (INF) neuron model [20–22]. Simulation of event-based models advances with each event rather than by a predetermined timestep. The resultant low computational burden means that INF neurons are often the first choice when simulating large networks to study network structure and network dynamics [23] but the simplified neuron model reduces the biological relevance in the context of living nervous systems by omitting biophysical detail of the neuron unit.

Event-based modeling need not, however, be limited to simplified neuron models. Here, we introduce an event-based framework which uses event-triggered functions as a means to model neuron responses. In our framework, on-event functions are user-defined functions called at each synaptic event that receive the list of recent synaptic inputs (and possibly cellular spike times) and output the time-to-next-spike, or infinity if there is no next spike. These functions model the neuron as an input/output (I/O) relationship of synaptic events and action potential responses, instead of modeling the neuron’s membrane voltage and latent state variables as in conductance-based modeling. Instead of focusing on any particular function, we approximate the error inherent to the event-based modeling approach and test the assumption that limited numbers of input events can reproduce the conductance-based model responses. Any specific function (e.g. from machine learning) will introduce additional error. We demonstrate that the spiking neuron behaviors are reproducible with a strictly event-based system of a small number of the most recent stimuli. We test this approach with point cells (various parameterizations of Hodgkin-Huxley (HH) [24] and the Wang-Buzsáki (WB) [25] model) as well as a morphologically and biophysically detailed cell model with active dendrites [26] to explore the relationship between known history and deterministic behavior for both single spikes and sustained activity. We further show that for our test cells the distribution of cellular state variables may likewise be highly constrained by a relatively small sampling of the recent event space.

## Methods

### Conductance-based neuron modeling

**Fig 1:**
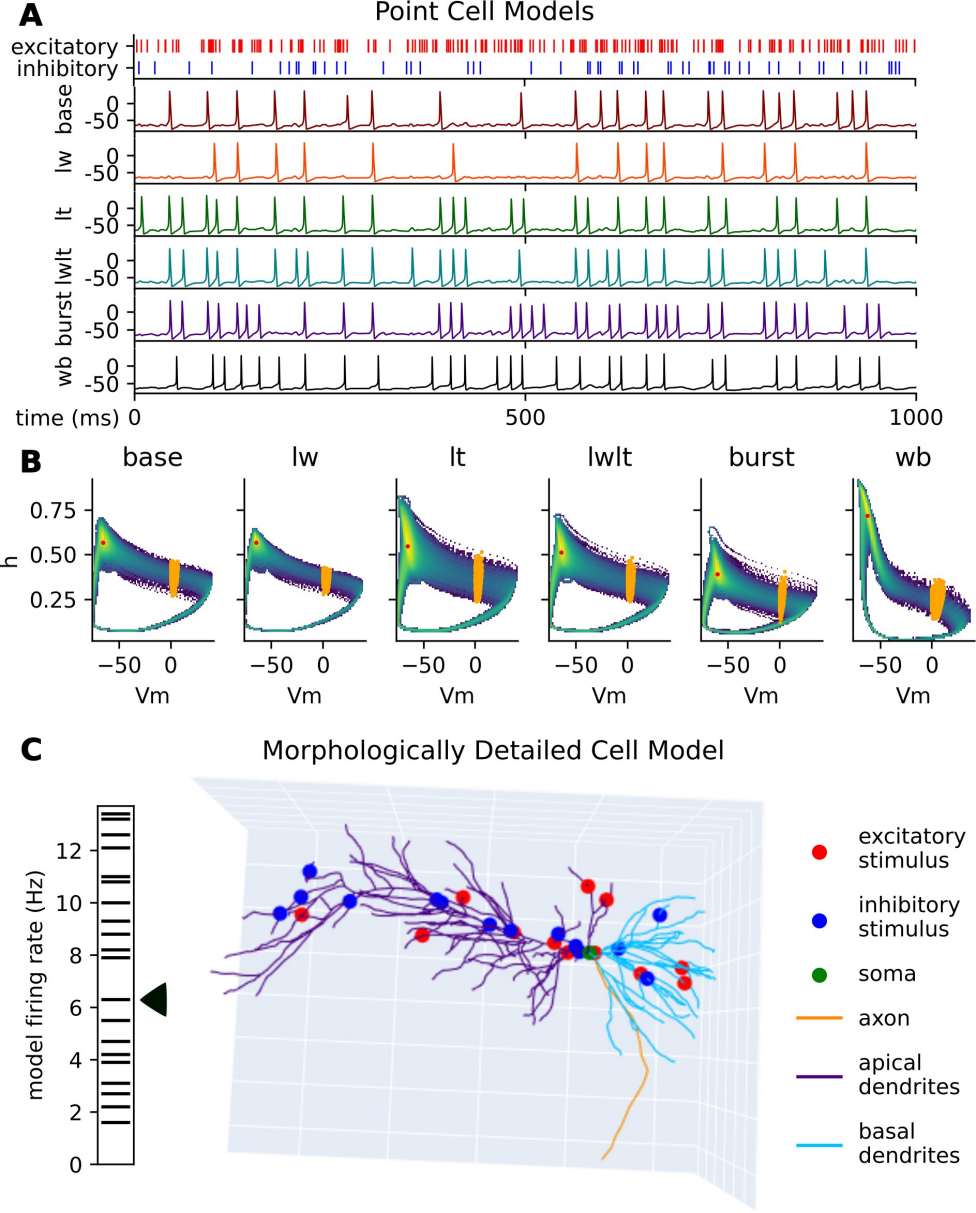
Point cell model behavior. **(A)** Example membrane potential traces for the Wang Buzsáki neuron model (wb) and five synaptic parameterizations of the Hodgkin-Huxley neuron model (Table 1) demonstrate different spiking behaviors, ranging from a low firing rate to the baseline behavior to a more bursty dynamic, given the same synaptic stimuli event pattern. **(B)** Density maps of the state space (*Vm* and *h*) of the HH and WB neuron models. Indicated are the models’ median state variable frame (red) and spiking state variable frames (orange). **(C)** 3D render of the morphologically detailed CA1 pyramidal cell model with the excitatory and inhibitory synapse locations, soma, and morphological regions indicated. The specific synapse locations shown here is one of twenty used in this paper, and exhibits a mean firing rate of 6.3 Hz, in the middle of the range for all firing rates from the 20 synaptic arrangements shown on the left of the figure.

All continuous-time conductance-based neuron simulation was accomplished using the NEURON (version 8.2) simulation environment [27]. We simulated single compartment neurons with HH dynamics [24], whose synaptic inputs were parameterized to exhibit five distinct behaviors (Table 1). Four of these parameterizations—Base, LW, LT, and LWLT–combine low and high stimuli weight parameterizations with slow and fast synaptic time constant parameterizations. The fifth model parameterization exhibits bursting behavior similar to type II bursting (Fig 1A). We simulated single compartment neurons with WB dynamics [25] using the NEURON implementation in ModelDB entry 26997 (https://modeldb.science/26997). For all models, stimuli events trigger exponentially decaying synaptic currents implemented with NEURON’s ExpSyn mechanism. Without loss of generality, we define the timing of an output spike as the moment during which the membrane potential crosses 0 mV from below within the single compartment for point cells and within the axon segment adjacent to the soma for the morphologically detailed CA1 pyramidal cell model.

**Table 1:** Cell model synaptic parameterizations. The five synaptic connection parameterizations tested for single compartment cell models with HH dynamics and one for the Wang-Buzsáki single compartment cell model produce distinct spiking behaviors that span short and long time dynamics, low and high reactivity to stimuli and spiking and bursting behavior. Additionally, the 20 synaptic arrangements for the morphologically detailed CA1 pyramidal cell model exhibit a range of firing rates from 1.6 Hz to 13.4 Hz.

	Excitatory synaptic weight	Inhibitory synaptic weight	Excitatory synaptic time constant	Inhibitory synaptic time constant	Average spike frequency
Base	0.0002	0.0005	2ms	6ms	22.14 Hz
Low Weight (lw)	0.00015	0.0002	2ms	6ms	13.94 Hz
Long Tau (lt)	0.0002	0.0005	10ms	40ms	17.62 Hz
Low Weight Long Tau (lwlt)	0.00015	0.0002	10ms	40ms	23.10 Hz
Burst	0.0001	0.0005	40ms	20ms	30.07 Hz
Wang-Buzsáki (wb)	0.00003	0.00005	2ms	6ms	25.03 Hz
CA1 pyramidal (Morse et al 2010)	0.0015	0.003	2ms	6ms	1.6–13.4 Hz

We simulated a 3D CA1 pyramidal neuron using the morphology and membrane properties from ModelDB entry 87284 (https://modeldb.science/87284) [26]. In this model, all compartments have active ion channels. The spatial compartment sizes are chosen as in the previous paper according to the d_lambda rule [28] to a fraction of the local AC length constant computed from the biophysical properties of the model. We placed 15 excitatory and 15 inhibitory synapses along the dendrites of the model in 20 different spatial arrangements (Fig 1C). Each synapse received an independent stimuli stream generated with a Poisson process with an expected interval of 40 ms.

Using a 100,000 ms simulation of each of the point cell model synaptic parameterizations and a 1,000 ms simulation of each of the morphologically detailed cell model synaptic arrangements, we generated random state variable frames and spiking state variable frames to be used as initializations in our experiments. Random state variable frames are the state variables Vm, h, n and m of the HH model, Vm, h, and m of the WB model, and all state variables of all segments in the CA1 pyramidal cell model sampled at random times in the simulation. 10,000 random state variable frames were generated for each synaptic parameterization of the HH models, the WB model, and each of the synaptic arrangements of the CA1 pyramidal cell model. Spiking state variable frames are the subset of all frames which occur at the onset of an output spike (Fig 1B). Median state variable frames represent the model’s median subthreshold state. To find the median state variable frames, we calculate the median value for each normalized variable and use the corresponding observed state variable frame with the minimum distance from those median values (red points in Fig 1B).

### On-event simulation framework

To test the effect of varying event-based input encodings on neuron responses we developed an on-event simulation framework within NEURON that decouples event responses from simulation details like initial conditions, model states, and prior event history. This decoupling allows us to present the model with precise amounts of stimuli events from which to generate responses in order to study the model’s tolerance to ambiguity prior to its recent history. Our on-event simulation uses NEURON’s discrete event simulation and manages the timings and types of simulation events (stimuli and output spikes); no latent state variables or membrane voltages exist in this simulation instance. Stimuli events trigger parallel on-event functions which are used to model the cell’s response given its recent event history. These functions then communicate the resulting responses back to the on-event simulation. The on-event function with n events, triggered by event e_i_, receives the events e_i-n_, e_i-(n-1)_, …, e_i-1_ as its inputs. Here, each event e_i_ is represented as the event’s time and input type. The point cell models in this paper incorporate the input types as excitatory stimuli, inhibitory stimuli, and output spike events (e.g. the neuron’s own action potentials). The morphologically detailed models in this paper incorporate a unique input type for every unique input location along the cell’s morphology. The framework does not have a requirement for the number of input types in the on-event function inputs; the number of input events as well as the input types are user-defined. The on-event function computes the next-spike-time (NST)—the simulation time until the next output spike event, which is set to infinity if the simulation is predicted not to spike provided no further input. Given a predicted finite NST, if the main simulation receives no other input before the predicted spike, the spike is triggered and recorded. If, however, a stimulus event occurs prior to the predicted spike, the spike is ignored, and the model’s behavior is recalculated using a new on-event function with the newly generated recent event history. On rare occasions, multiple stimuli may lead to the same predicted spike time; only one output event is generated at a given time point. The framework is designed such that this on-event function is an interchangeable Python function, and can be flexibly defined by the user.

### Limited event-based input encoding experiment

The limited event-based input encoding represents the total history a cell uses for generating its response as the timings of the last *n* events in the cell’s history. Incorporating this encoding in a neuron model constrains the total size of the input space (all historical events and initial conditions to only its *n* most recent historical events) without changing the size of the response space (the NST).

We measured the error introduced by the limited event-based input encoding using a parallel instance of NEURON that runs a short conductance-based neuron model as our on-event function within the on-event simulation framework. This is achieved by running a short, parallel, NEURON simulation of the original conductance-based cell model upon each trigger of the on-event function (i.e., on each synaptic event). The parallel cell model’s initialization and input depend only on the events ei-(n-1), ei-(n-2),…,ei of the on-event function. These on-event instances are initialized with one of 10,000 random state variable frames to account for variability in the model’s initial conditions. When the first event is an output spike, the set of random state variable frames used to initiate the on-event instance is constrained to a spiking state variable frame (Fig 1B). On-event instances run for the duration of the recent event history plus 20 ms for the point cell models and plus 50 ms for the morphologically detailed cell models. The NST is the time between the last input event and the next spike in the simulation; the first spike of the extended 20 or 50 ms; or infinity if there is no resulting spike. The on-event instance communicates the corresponding NST to the main simulation which advances to the time of the next stimuli or to the time of the predicted spike, whichever comes first.

We evaluate the effect of the limited event-based input encoding on neuron models in two primary contexts: how it affects the presence and timing of single response spikes and how it affects the neuron model response over time.

To assess the extent to which the number of events (*n*) in the limited event-based input encoding alters the presence and timing of single response spikes, we simulated numerous cells with varying amounts of the same partial history. First, 1,000 unique stimuli patterns with 50 combined excitatory and inhibitory stimuli were generated as the full histories. For each value of *n* in 3, 4, … 50, we used the *n* most recent events of each of these 1,000 full histories, as a partial history. Each of these partial histories was run in 1,000 simulations each initialized with one of the 1,000 random state variable frames. This was done for all 5 synaptic parameterizations of the HH point cell and the WB point cell models. The resulting 288 million simulations were used to analyze the robustness of the event-based framework to variations in the initial conditions given differing values of *n* inputs in the recent event history.

We consider metrics to describe the consistency of spike presence, spike placement, as well as categorical descriptions of a response set describing its level of determinism. We introduce the metric of spike prediction coherence to measure the degree of consistency in the presence of spikes in the set of responses generated from randomized initializations. Given a stimuli pattern and *k* random state-variable frames as initial conditions, a fraction of their respective *k* simulations will respond with a spike. The spike prediction coherence measures the probability of pairwise agreement of spike presence given any two of the *k* simulations (ratio of spiking responses squared plus the ratio of non-spiking responses squared). To qualitatively evaluate the evolution of responses observed as the recent event history changes, we consider a set of responses as deterministic non-spiking if less than 1% of the responses spike, as non-deterministic spiking if 99% of the responses spike and the standard deviation in spike timings is greater than 0.1 ms, as deterministic spiking if 99% of the responses spike and there is less than 0.1 ms standard deviation in the timings, and as non-deterministic if it meets none of the other criteria.

We also analyzed how this input encoding would affect the neuron model response over time. To do so, we first generated ground truth responses of the conductance-based models for comparison. These included 10,000 ms simulations for each of the 5 synaptic parameterizations of the HH point cell models, the WB point cell model, and each of the 20 synaptic arrangements of the morphologically detailed cell model. Then, using the on-event simulation framework with the original conductance-based neuron model as the on-event function, we ran extended simulations of the neuron models with the limited event-based input encodings. We tested using 3, 4, …, 50 recent events with and without incorporating the timing of the last output spike for the point cell models. When no recent event was an output spike, we started each simulation from the median state variable frame. Output spikes were modeled in the on-event function by initializing the simulation with a spiking history rather than with the median history. Thus, necessarily, the incorporation of the last output spike in the input encoding truncates the encoding if there were stimuli events prior to the last output spike. The limited event-based input encodings included n in 1, 2, …, 10 recent events from each of the 30 synapse locations on the morphologically detailed model, totaling 30–300 stimuli events. With the resulting spike trains of the ground truth simulations and input encoding simulations, we compared the observed continuous firing rates and interspike interval (ISI) distributions, as well as the distance between the pair of spike trains calculated using the van Rossum metric [29] as implemented in the Elephant electrophysiology toolkit [30].

### State variable reconstruction

We measured the ability to reconstruct the state variables of a simulation provided only the input events by measuring the extent to which the state variables of the models varied between two simulations with identical inputs but different initial conditions. For each of the 5 synaptic parameterizations of the HH point cell and the WB point cell, we simulated 5,000 pairs of simulations with matching input events for 100 ms. For each of the 20 synaptic arrangements of the morphologically detailed cell model we simulated 250 pairs of simulations with matching input events for 200 ms. The membrane voltage of the point cells and of every segment of the morphologically detailed cells were recorded for analysis.

## Results

To examine the viability of the on-event simulation strategy as an effective alternative to conductance-based modeling, we ran three sets of analyses. First, we explored the degree to which action potentials constrain neuron states and act as natural barriers of information in neuron input history. Such information constraining events in the model act as evidence that the response of a cell is probabilistically determined by a recent subset of its input stimuli as they can help diminish propagating error. We showed that the possible responses are well characterized by the timings of the *n* most recent events. The value of *n* is model-specific and depends on e.g., synaptic decay time constants and gating variable time constants. We found that models with a variety of different stimuli, like the varying placement of synapses along model morphology, benefit from categorically distinguishing these stimuli but do not show greatly increased demands in the total number of events needed proportionate to their magnitudes higher state variable count and model complexity. Finally, we demonstrated the interoperability of conductance-based and event-based modeling by testing the extent to which the state variables of point and morphologically detailed models could be reconstructed from event timings only.

### Action potentials act of information bottlenecks in simulation

Action potentials in the HH system produce a stereotyped, cyclic pattern in its phase plane. The density around this phase plane is non-uniform, with depolarization displaying more variation and thus lower density than repolarization and refractory periods (Fig 1B, Table 2). Because of their canonical shape, we considered action potential timings alongside stimuli timings as events in an event-based input encoding model.

**Table 2:** Comparison of membrane potential variance conditionally around spike events. Observed variance in membrane voltage of the entire simulation (baseline Vm variance) compared to that conditionally centered around the spike. Note that the exponents in the scientific notation are different in different rows.

	Baseline Vm variance	Spike centered minimum Vm variance (mV^2^)	Percentage of baseline variance at spike	Time of peak variance before spike	Time of peak variance after spike
Base	1.84 x 10^−2^ mV^2^	3.92 x 10^−6^ mV^2^	0.02%	-16.7 ms	18.9 ms
LW	1.25 x 10^−2^ mV^2^	2.47 x 10^−6^ mV^2^	0.02%	-17.7 ms	18.9 ms
LT	1.44 x 10^−2^ mV^2^	1.73 x 10^−5^ mV^2^	0.12%	-13.9 ms	17.2 ms
LWLT	1.78 x 10^−2^ mV^2^	1.08 x 10^−5^ mV^2^	0.06%	-15.1 ms	15.7 ms
Burst	1.74 x 10^−2^ mV^2^	1.80 x 10^−6^ mV^2^	0.10%	-14.0 ms	15.5 ms
wb	8.04 x 10^−3^ mV^2^	2.40 x 10^−5^ mV^2^	0.30%	No discernable peak	No discernable peak

**Fig 2:**
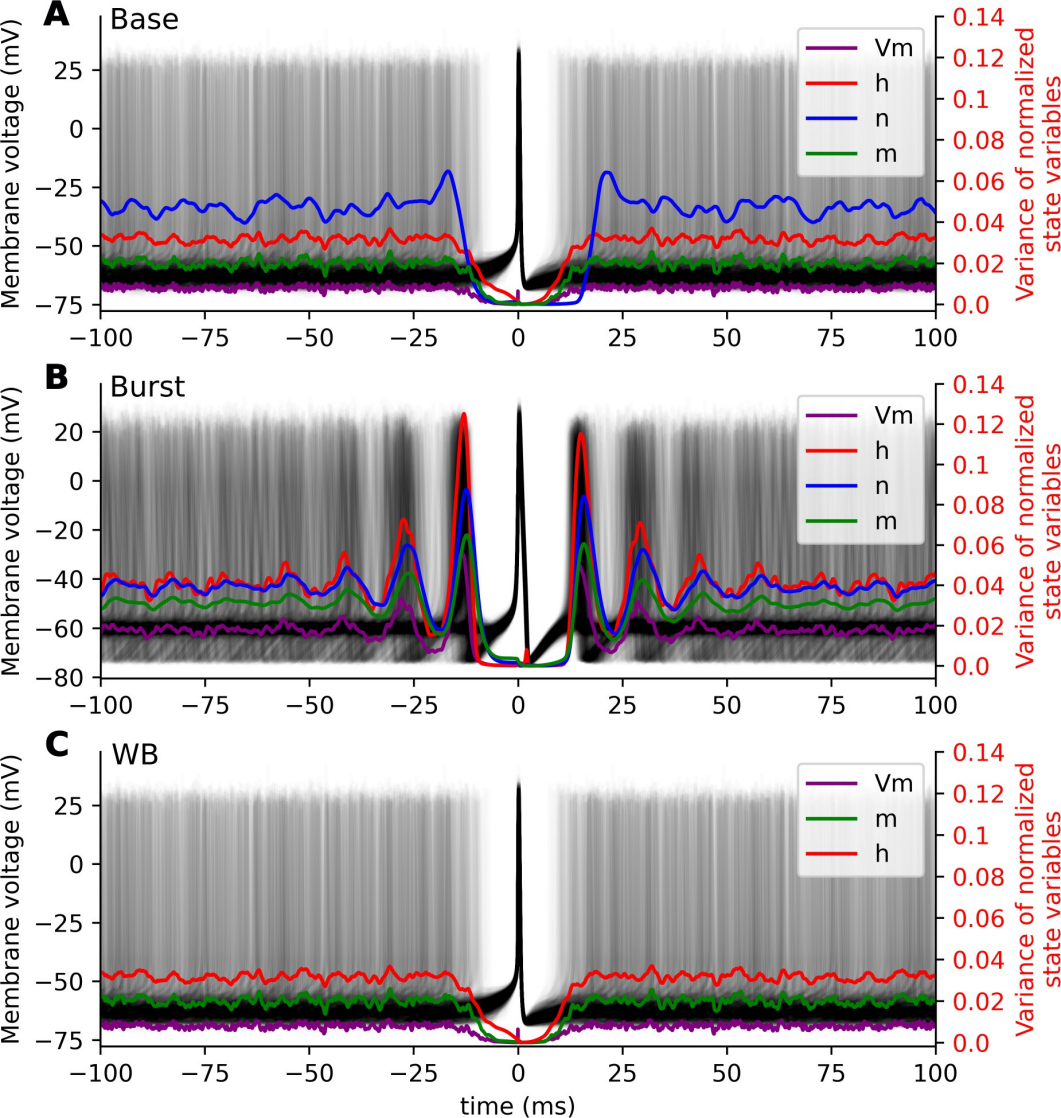
State space variation centered on spike events. 1,000 membrane voltage traces centered at a spike (black lines) and the observed variance in 40,000 simulation windows of normalized Vm (purple), h (red), n (blue) and m (green), for the base **(A)** and burst **(B)** synaptic parameterizations of the HH point cell model and the WB model **(C)**. In all cases, the variance in state variables is significantly reduced relative to baseline for several milliseconds before and after the centered spike.

To test the extent to which action potentials constrain a cell’s state, we measured the spike-triggered-variance in the observed state variables. We found a consistent and large reduction in variance conditionally around spike events for all the measured point cell models when compared to their respective state variable variance observed across the entire simulation (baseline Vm variance) (Table 2). Models with longer time kinetics (LT, LWLT, and Burst) showed less of a reduction on the baseline variance (Table 2). The canonical form of the action potential resets the cells’ states and dramatically reduces the effects of previous stimuli on state variables for the HH model and the WB model (Fig 2). The variance of state variables was elevated immediately before and after the spike for the HH point cells at the approximate interspike interval, but no such pattern was observed for WB (Table 2, Fig 2).

### Model determinism as a function of the number of inputs in recent event history

**Fig 3:**
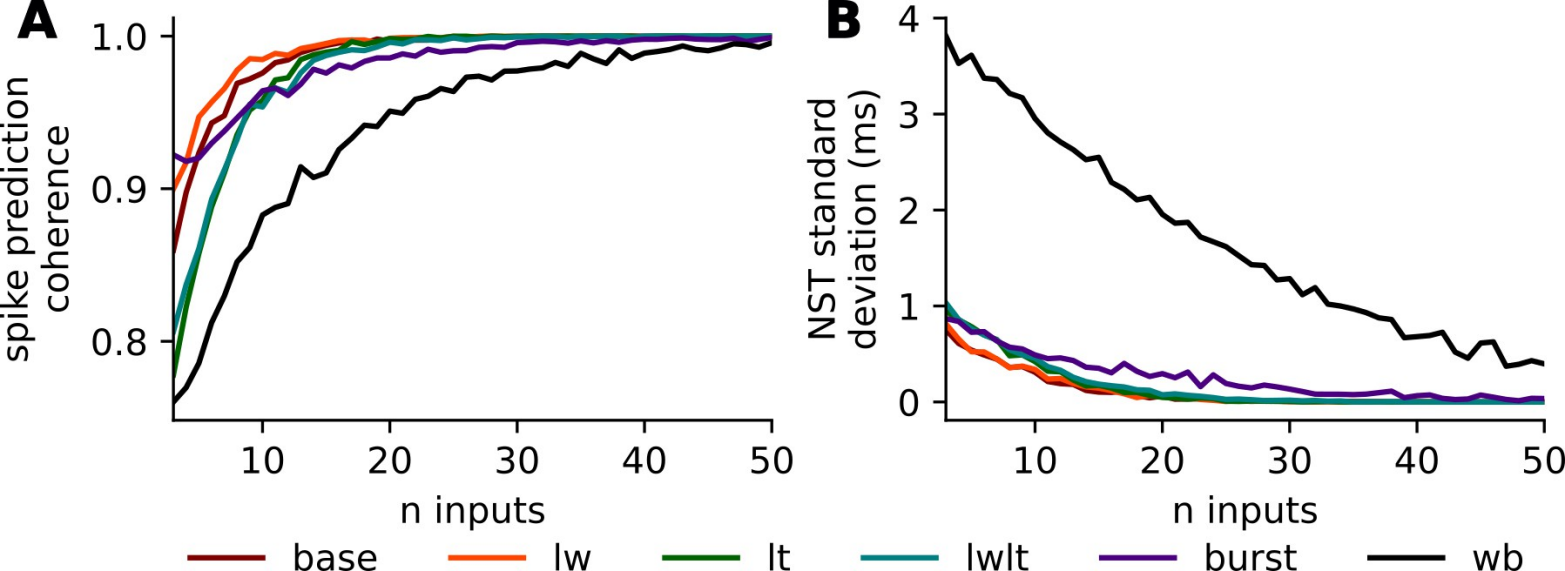
Spike prediction coherence and NST standard deviation as a function of size of recent event history. Two measures used to evaluate the determinism of spiking responses in point cell neuron models, given randomized initializations and recent event history. Spike prediction coherence **(A)**, is calculated as the ratio of spiking responses squared plus the ratio of non-spiking responses squared. NST standard deviation **(B)** is calculated from the distribution of observed timings of spiking responses across multiple trials. Both measures demonstrate generally increasing determinism with more inputs, with spike prediction coherence converging faster than spike timing.

As the number of inputs in the recent event history increases, the spike prediction coherence of the responses increases and the NST standard deviation decreases: i.e., the distribution of responses tends towards determinism. This trend plateaus after approximately 15 inputs for all synaptic parameterizations of the HH point cell to varying degrees (Fig 3). The Wang-Buzsáki model arrives at comparable spike prediction coherence after roughly 50 inputs indicating that its response is sensitive to a larger extent of its input history. The standard deviation in the timing of the observed spikes (NST standard deviation) for the HH models approaches zero, but requires roughly 25 inputs in the recent event history, approximately 10 more input stimuli than the spike presence prediction (Fig 3). This indicates the placement of the spike timings is more sensitive than spike presence to the exact state of the cell and requires more event history to accurately predict. The NST standard deviation for the Wang-Buzsáki model displays the same trend as the HH models but at a significantly slower rate, such that even after 50 inputs there remains noticeable variability in the NSTs.

The Base and LW synaptic parameterizations and the LT and LWLT slow synaptic parameterizations have similar values for both the spike prediction coherence and the NST standard deviation, suggesting that the synaptic decay time constant is more influential than the synaptic weight constant on the variance observed in the responses. The plateau observed in both the spike prediction coherence and the NST standard deviation for all synaptic parameterizations indicate a high level of response determinism, especially for the HH cell model, and provide evidence that the model states are accurately encoded with the recent event history input events (Fig 3).

**Fig 4:**
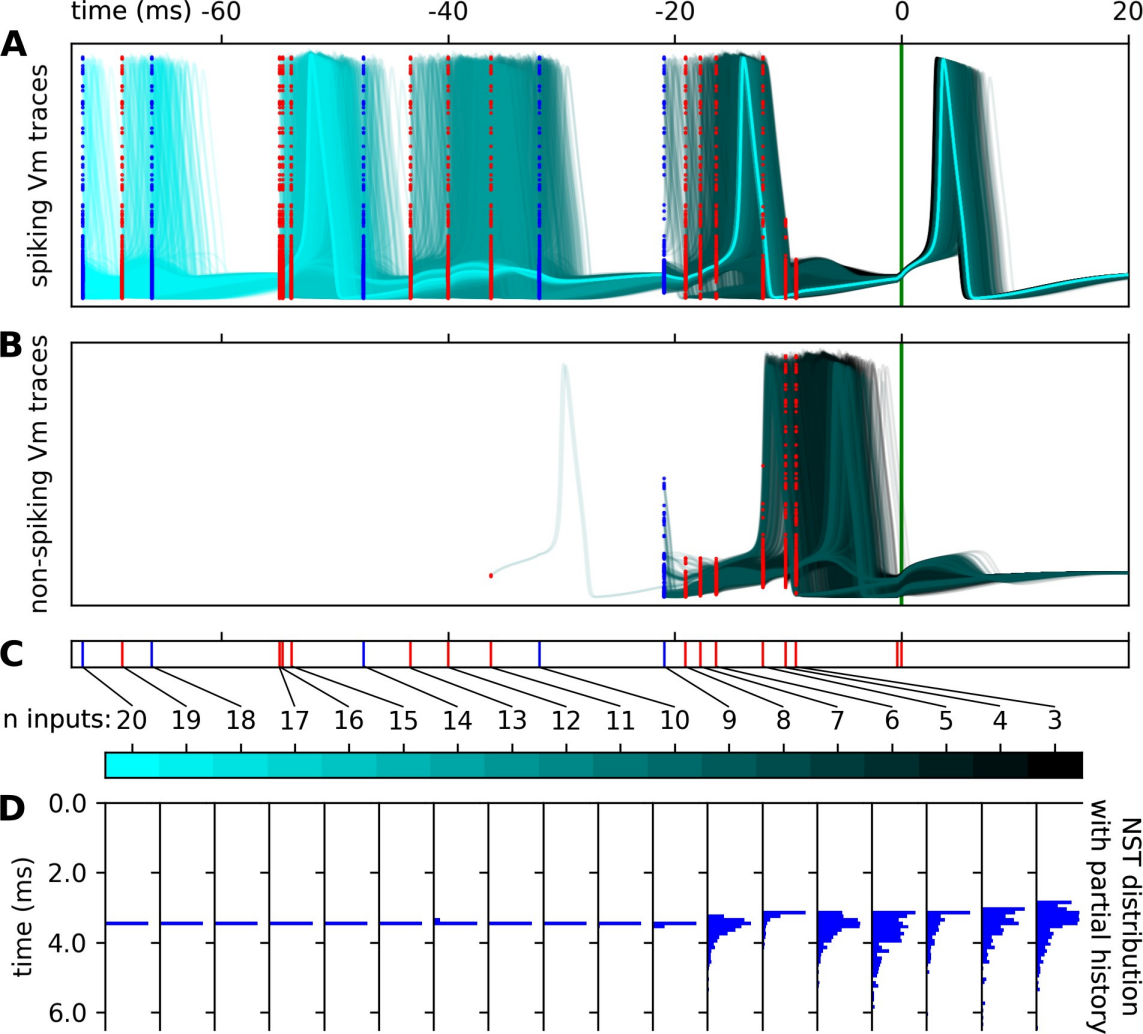
Evolution of spiking behavior as a function of number of inputs in input encoding for a given stimuli pattern. The Vm traces of spiking **(A)** and non-spiking **(B)** simulations with 20 (cyan) to 3 (black) input stimuli events in the recent event history for a single stimuli input pattern **(C)**. Initializations of the simulations of these Vm traces indicated excitatory input stimuli events (red dots) and inhibitory input events (blue dots) correspond to the **(C)** input event raster. **(D)** The NST distribution corresponding to the specific stimuli events in **(C)** as histograms. The NST distribution histograms are independently scaled for visual clarity, but have 0.67 spiking ratio for 3 inputs, 0.34 for 4 inputs, 0.29 for 5 inputs, 0.39 for 6 inputs, 0.78 for 7, 0.87 for 8, 0.82 for 9, and 1.0 for 10–20 inputs. This example highlights the specific stimulus instance responsible for collapsing the distribution of responses to a deterministic state.

**Fig 5:**
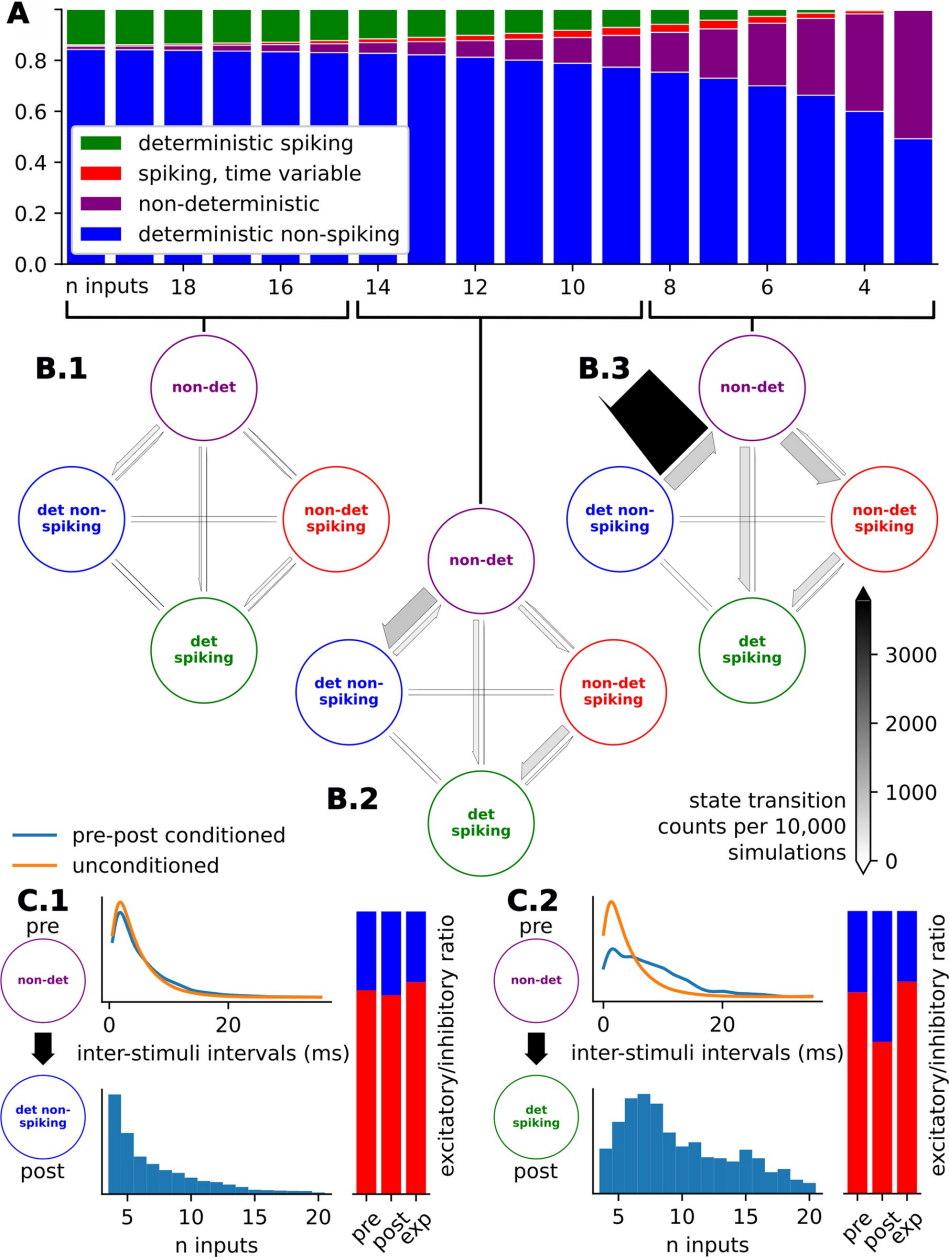
Response determinism of event-based input encodings with varying input history. **(A)** Ratios of response distribution categories as the number of input events in the input encoding, *n*, increases shows a general increase in determinism when more inputs are known. **(B)** Phase transition diagrams showing the transitions between response distribution categories for **B.1)** high values of n (n in 15, 16, … 20), **(B.2)** medium values of n (n in 9, 10, … 14), and **(B.3)** low values of n (n in 3, 4, … 8). **(C)** Input conditions responsible for phase transitions between **(C.1)** non-deterministic to deterministic non-spiking and **(C.2)** non-deterministic to deterministic spiking phase transition. Input conditions include 1) inter-stimulus interval of the stimulus responsible for the transition (blue) compared to the unconditioned inter-stimulus intervals (orange), 2) histogram showing the relative presence of the specific transition category in relation to the number of input events, and 3) the ratios of stimuli types (excitatory: red and inhibitory: blue) of stimulus just before the transition (pre), of the stimulus responsible for the transition (post), and the expected, unconditioned stimuli type ratios (exp).

We show the responses for a specific input stimuli stream given a variety of initial conditions and an evolving number of stimuli in the input encoding as an example (Fig 4). We characterized the distributions of responses given limited input encodings of a multitude of stimuli patterns categorically as deterministic spiking, deterministic non-spiking, non-deterministic spiking, and non-deterministic and investigate the specific conditions in the stimuli patterns that force the response distributions to switch between these categories (Fig 5A).

For the stimuli pattern in Fig 4, uncertainty on the presence of a spike persists up to the inclusion of the 10th input event, when the spike presence ratio goes from 0.82 (n = 9) to 1.0 (n = 10). This represents a switch from the category of non-deterministic to deterministic spiking, observed in the drastic change in NST distributions between the inclusion of this stimulus (Fig 4D). What is notable about the 10th input, the input responsible for this categorical change, is the large interval between it and the previous, 9th, stimulus. The tendency for long inter-stimuli-intervals corresponding to switches to the deterministic category is reflected in the conditional difference in inter-stimuli-intervals for the stimuli responsible for non-deterministic to deterministic switching compared to the unconditional stimuli intervals (Fig 5C.2). Likewise, the response distributions switch from non-deterministic to deterministic spiking conditionally more frequently when the post stimulus is inhibitory (Fig 5C.2).

The vast majority of deterministic spiking and deterministic non-spiking response distributions remain that way given as *n*, the number of input stimuli in the input encoding, increases. Further, the total number of transitions slows down as *n*, the number of stimuli in the input encoding, increases (Fig 5B). The observation that non-deterministic spiking response distributions tend to transition to deterministic spiking response distributions further emphasizes that spike timing prediction as a task requires more input information than spike presence prediction (Fig 5B).

The state transition diagrams track the changes in the categorical set of responses observed from a neuron in our event-based framework (Fig 5B). As the framework incorporates more input history, in the form of additional stimuli events, the distribution of responses narrows. As shown in the state diagrams, additional input history makes the response distribution tend towards determinism, or more agreement in the response. Once a stimuli pattern has been identified as driving a deterministic spiking (or non-spiking) result, adding more known prior stimuli rarely changes this prediction, suggesting that we are choosing sufficiently many initial conditions to make this determination. Further, the total transitions become more infrequent as *n*, the number of stimuli in the input encoding, increases (Fig 5B). The observation that non-deterministic spiking response distributions tend to transition to deterministic spiking response distributions indicates that spike timing prediction as a task requires more input information than spike presence prediction (Fig 5B).

### Event-based models with limited history reproduce realistic spike-trains

**Fig 6:**
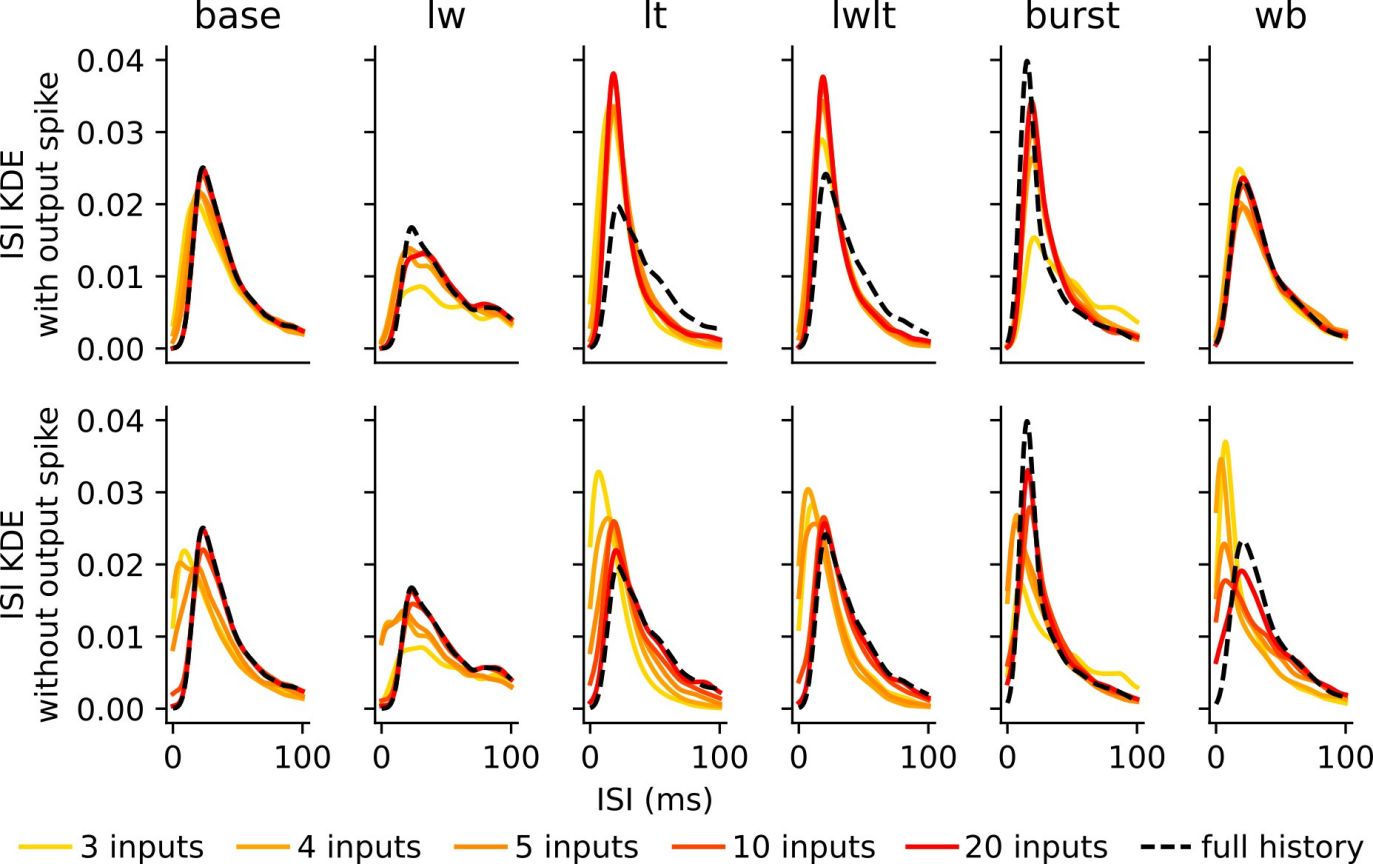
Evolution of ISI distributions as a function of number of inputs in input encoding. Observed inter-spike-interval distributions of spike trains with limited input encoding, shown as *n* increases, for the encodings with **(top)** and without **(bottom)** prior output events. These ISI distributions are compared to the ISI distribution of the point cell models without the input encoding (full history). ISI distributions generally match the full history more closely with more input history. Including the time of the last output spike improved baseline distribution matching when few input times are known, but impaired the matching for the LT and LWLT cases when *n* is larger.

To evaluate the impact of limited input encoding in a full neuron simulation, we compared the observed spike trains and ISI distributions of the HH and WB models with and without the limited input encoding. The ISI distributions of the limited input encoding models that do not include the prior output spike as an input event, include unrealistic ISIs for low numbers of prior events (*n* < 5) suggesting this number of inputs is often incapable of accounting for the neuron’s refractory period (Fig 6) which does not allow two action potentials to occur is short succession. By contrast, including the time of the most recent prior output spike event greatly reduced the presence of unrealistically small ISIs (Fig 6). For large *n*, including the output spike as a start point reduces the replicability of the ISI distribution, especially for the synaptic parameterizations with long time constants as its inclusion often truncates the size of the input encoding (Fig 6).

**Fig 7:**
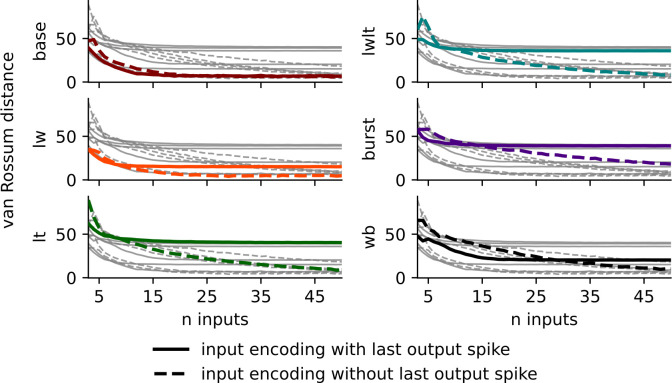
Spike train replicability of HH and WB models with event-based input encodings as a function of number of inputs. van Rossum distance between observed spike trains from the neuron models with and without the event-based input encoding for each of the five HH model parameterizations and the WB model. Shown are the distances for encodings which include the output spike (solid lines) and encodings which do not include output spike (hashed line). When very limited historical stimuli information is available, including the time of the last output spike reduced van Rossum error, but with larger amounts of stimuli history, for all but the baseline case the van Rossum error was lower on models generated without incorporating the last output spike time.

We compared the behavior of the neuron models with and without the input encoding using the van Rossum spike train edit distance metric [29] (Fig 7). The van Rossum distance decreased between the spike trains as the number of inputs in the encoding increased for all series, but converged to zero edit distance more quickly for the Base and LW parameterizations. The output spike train of the Burst parameterization proved to be the hardest to replicate, with a considerable van Rossum distance even after 40 input stimuli. For low n, the inclusion of the output spike in the encoding provides a more rapid reduction in van Rossum distance due to its elimination of unrealistically small ISIs (Fig 7), but plateaus after roughly ten inputs (Fig 7). Including the output spike necessarily omits all synaptic information prior to the respective spike in our experiments; we could not initialize the on-event simulations randomly and ensure a spike event at a given time. This constraint means that our analysis truncates stimuli history to the last output spike, meaning no further improvement is possible with additional stimuli events.

### Limited input encodings for morphologically detailed neurons require categorically distinct stimuli

Morphologically detailed models are discretized into many spatial compartments, each of which, depending on the specific model, can be a potential location for synaptic input. In the CA1 pyramidal cell model, all dendritic segments were considered when placing each of the 30 synapses. To account for this increased complexity within event-based modeling, we treat each distinct input location as an independent stimuli type in the same way the event-based point cell model distinguishes excitatory from inhibitory stimuli. Thus, the input-encoded morphologically detailed neuron with 15 unique excitatory and 15 unique inhibitory input locations receives the n most recent stimuli from all 30 sources.

**Fig 8:**
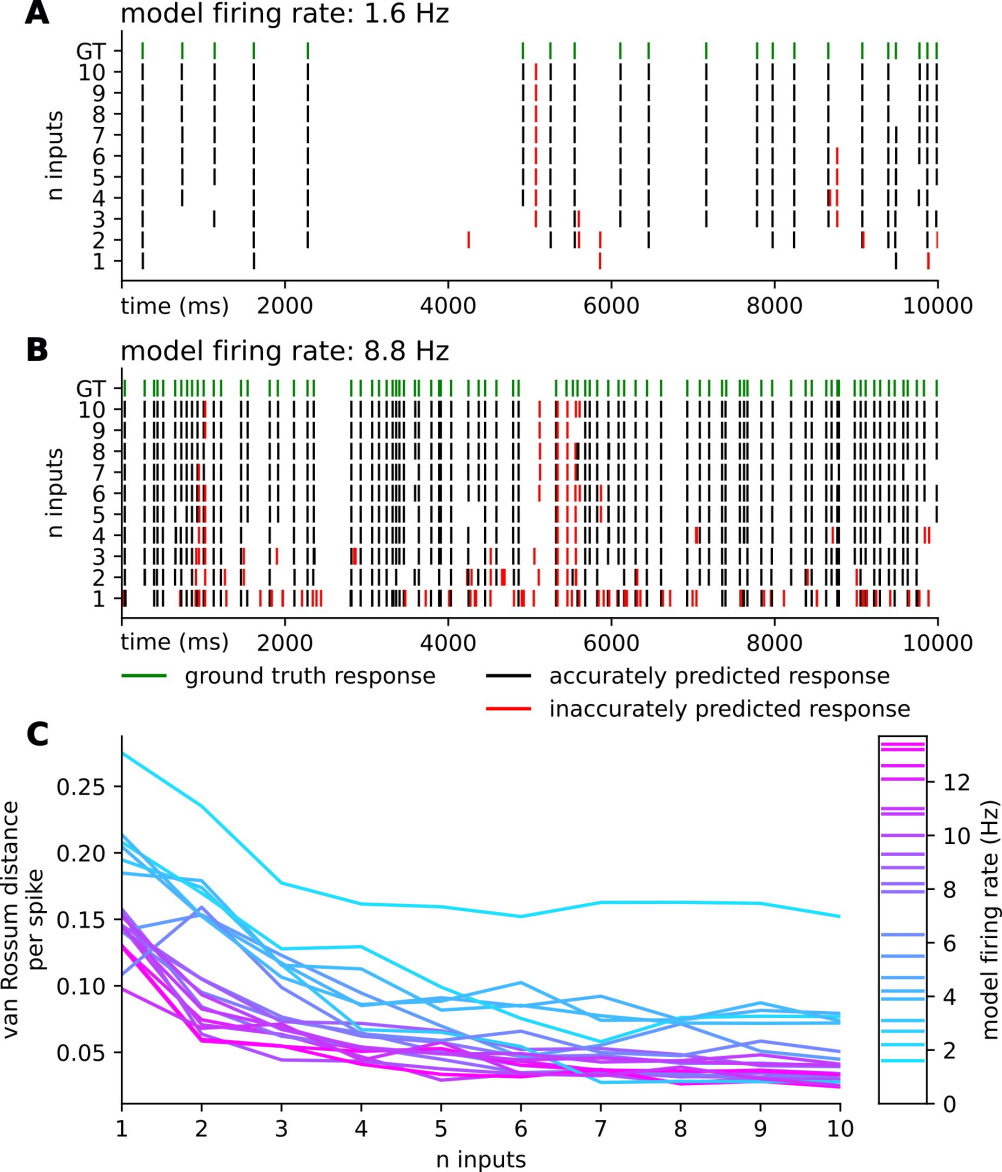
Spike train replicability of CA1 pyramidal cell model with event-based input encoding. Raster diagrams showing the simulated responses of a low **(A)** and a high **(B)** firing rate synaptic arrangement morphologically detailed conductance-based CA1 pyramidal cell model as the ground truth (green) compared to the replicated responses of the event-based neuron model with recent event histories of size 1–10 input events per synapse. Black ticks indicate spikes within 1 ms of ground truth spikes, and red ticks indicate spikes > 1 ms away from all ground truth spikes. Predicted spike times are broadly consistent when incorporating each additional input time, but become more accurate as more historical stimuli times are known. **(C)** van Rossum distance between observed spike trains per spike from the CA1 pyramidal neuron models with and without the event-based input encoding. Low firing rates show a slight increase in van Rossum error for the first few added inputs, but in general error decreased when additional input timings were included in the model.

As shown in Fig 8‘s raster diagrams and the van Rossum distance measures, the event-based framework better replicates the response of the analogous conductance-based simulation as it incorporates more input events. Cells with lower firing rates have proportionally higher van Rossum distance per spike (Fig 8C). Interestingly, the errors of the higher firing rate model appear to localize together (e.g., Fig 8B between 5,050 ms and 6,000 ms). We hypothesize that this is the result of propagating error going uncorrected for some time in the model and is a significant source of error especially for models with larger input encodings. Lastly, we notice that the errors of input encodings with larger values of *n* often share the same error. The incorrectly predicted spike at time t = 5075.28 in Fig 8B persists from n = 3 to n = 10. From our analysis we cannot conclude what extent of input history would be required to correct these types of error; more analysis is required to identify the conditions that result in these types of errors.

### Reconstruction of state variables from a strictly event-based model

As one of the key advantages of conductance-based models is the biophysical interpretation of their state variables, we examined the reconstructability of state variables across our six point cell models and the reconstructability of membrane potential (Vm) in the morphologically detailed model of [26]. To do so, we simulated each model from two randomly chosen spiking histories taken from the set of observed states, and measured the difference in the resulting membrane voltages. Results are reported based on 5,000 simulation pairs for each of the 6 point cells and 300 simulation pairs for each of the 20 synaptic arrangements of the morphologically detailed model.

**Fig 9:**
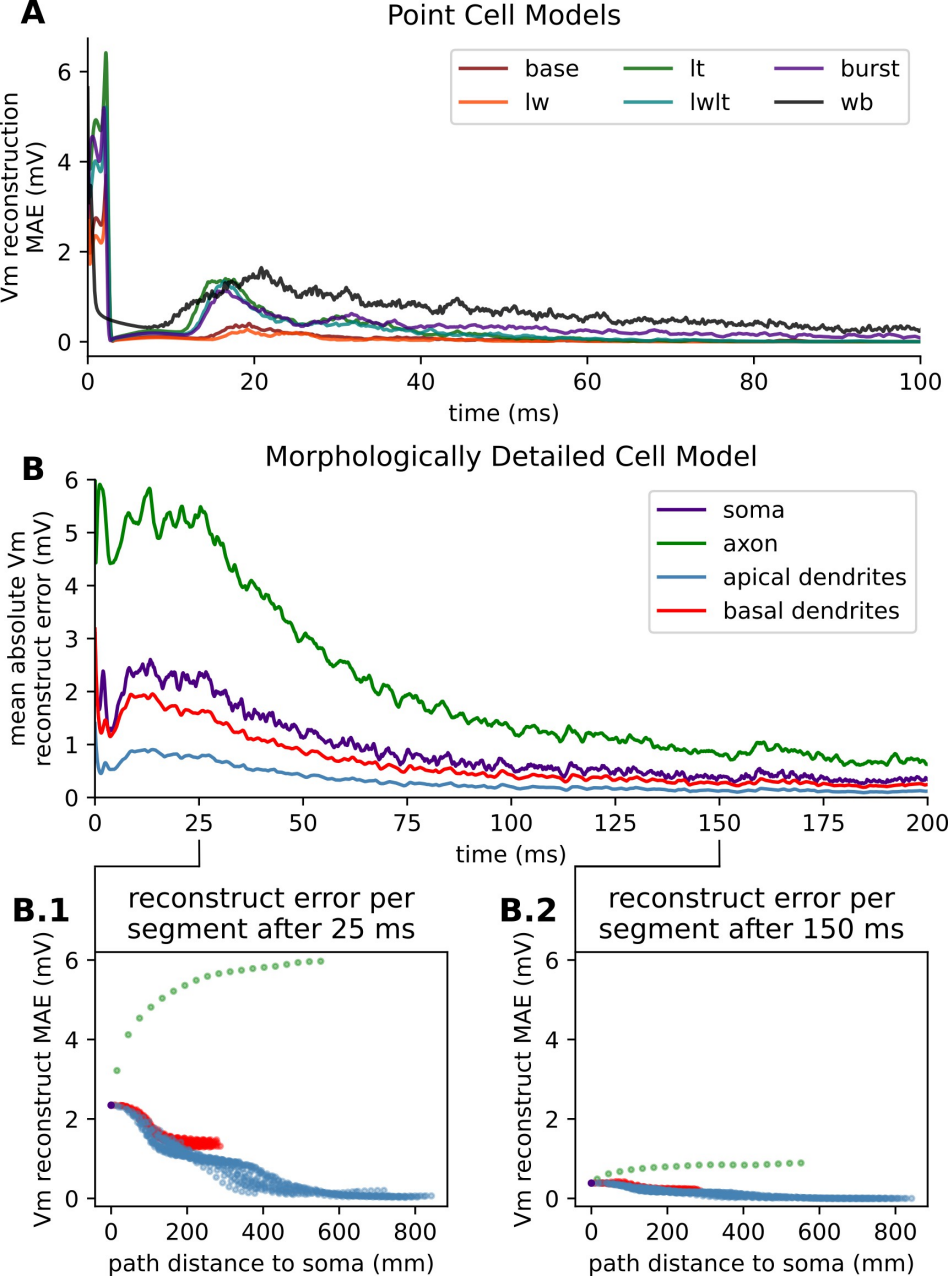
Vm reconstruction error as a function of time. **(A)** Mean absolute error of membrane voltage reconstructions for each of the five synaptic parameterizations of the HH point cell and the WB point cell. **(B)** Mean absolute error of membrane voltage reconstructions of each of the four neuron regions of the morphologically detailed CA1 pyramidal cell model. **(B.1)** Scatter plot comparing the mean absolute membrane voltage reconstruction error and the path distance to the soma for each segment in the model at 25 ms. **(B.2)** Scatter plot comparing the mean absolute membrane voltage reconstruction error and the path distance to the soma for each segment in the model at 150 ms.

For all tested point cell models, membrane potential mean absolute error (MAE) was highest within the first few milliseconds following a spike, likely due to discretization artifacts from detecting the timing of the spike. That is, in the span of the 25 μs of a typical NEURON advance, the membrane potential during the rising phase of a spike might increase by about 10 mV; this leads to comparable uncertainty in the true membrane potential at the reported spike time. The error in the membrane potential dropped and stayed low following the spike during the refractory phase of the cell, increasing at the end of the refractory period before decaying with further increases in time (Fig 9A). The base and low weight (LW) models showed the least error and the Wang-Buzsáki (WB) model error decayed the slowest.

We repeated the experiment reconstructing state variables following an action potential with the morphologically detailed cell model. The membrane potential errors were highest on average in the axon and soma which also generally have the highest membrane potentials due to their strong regenerative signaling. Apical dendrites showed the next lowest error, with basal dendrites the least (Fig 9B). At both 25 ms and 150 ms, the MAE in membrane potential decreases with distance from the soma for all compartments except the axon, where it increases with distance from the soma. The spatial pattern is broadly similar, but scaled, between the two time points (Fig 9B.1 and 9B.2).

## Discussion

### Event-based approach to modeling electrophysiological dynamics

In this paper, we introduce an on-event simulation framework that decouples the model’s responses from preexisting model states and demonstrate the feasibility of interpreting conductance-based models within this framework. Here we model a neuron as a function whose total history passes through a narrow input encoding, the times of a small number of its most recent input events, to output a response. With each extra piece of historical information, the distribution of possible and probable responses is further constrained. Prior work by [31] explored using the distribution of spike responses for representing populations of simplified Hodgkin-Huxley cells, but here our focus is on the possible responses of an individual cell and how that is constrained by the amount of information known. We test the extent to which the input encoding introduces error itself, separate from the specific modeling of the neuron response, by generating the response to the encoded input with a small instance of the conductance-based model in NEURON. This framework, however, is not limited to modeling responses in this way. Within this framework, the formalized mathematics and sub-threshold activity of the conductance-based model, or any model of neural response, can be folded into this on-event function without the need for solving state variables along a given timestep.

Encoding a cell’s state by its recent synaptic inputs is necessarily incomplete and thus serves as a source of error. Errors are, of course, intrinsic to the scientific process, ranging from measurement error, to modeling choices, and even the specific model of arithmetic used [32] and choice of numerical ODE solution procedure (e.g. Euler vs Crank-Nicholson vs Runge Kutta). We have shown for several specific point cell models that individual spike error and sustained spiking activity (as measured via the van Rossum metric) decrease with inclusion of additional input history, with the errors being smallest for the models we tested with short time constants. We showed similar results for the sustained simulation of a complex morphologically detailed neuron model. We predict these findings are applicable to many other such models. Use of our approach within network models will require examining how the individual spike errors affect the outcome measures (synchrony, firing rate, etc).

In the wake of the ever-increasing data on ion channels and cellular morphology, the complexity of conductance-based biophysical neuron models has grown greatly over the years. From the four state variables of the famous Hodgkin-Huxley model [24] to the cable theory models of dendrites using spatial compartments [33] to the routine use of morphologically detailed neuron models with upwards of 16,000 state variables each (e.g. [26]), computational neuroscience is consistently demanding more computational resources to meet its growing mechanistic complexity. We believe more generalizable approaches to event-based modeling can help alleviate some of the demands of biophysical neuron modeling by enabling data-driven techniques to bypass heavy mathematical computation.

### Evaluation of the event-based neuron model requires consideration of multiple contexts

We identified three major contexts for investigating the implications of event-based modeling—how it could affect the presence and placement of singular spikes, how well it can replicate sustained activity of a simulation of a neuron, and what it could change at the network level.

At the single spike level, conceptualizing the neuron as a function that responds to a given input with a consistent output lends itself to evaluating alternatives as predictive models. Metrics like spike presence prediction accuracy and spike time prediction error quantify how these two modeling schemas differ but fail to statistically measure the ambiguity in the distribution of responses. Instead, we used spike prediction coherence and standard deviation of NSTs to quantify the variance within a set of responses to the same input given differing levels of history. For an input stimuli pattern, if the discordance in responses is small enough, we say the outcome is functionally deterministic and the history is sufficient to characterize the response to that particular input. Thus, we categorically describe the observed sets of responses as spiking non-deterministic (high spike presence coherence but high NST standard deviation), non-spiking deterministic, spiking deterministic and non-deterministic. With this categorization we investigated the specific conditions that cause the resulting responses to become deterministic. Our approach to evaluating the single spike context for event-based simulation achieves the task of measuring the viability of this simulation strategy without framing it as a strict prediction task.

However, the error measured for individual spikes does not necessarily translate to potential errors on sustained cellular activity because of the nonlinearity of the system and potential for compounding error. Thus, we evaluated the model in the context of a neuron simulation over a given duration; that is, how well does the continued simulated response of the event-based neuron match that of the conductance-based neuron. We found the van Rossum spike train distance was an effective measurement that represents a veritable ‘edit-distance’ between the conductance-based neuron and event-based neuron responses given identical input stimuli. Van Rossum distance enabled us to evaluate our approach relative to conductance-based modeling using the only output of our framework; spike events.

Network level metrics like local field potential (LFP), synchrony, and firing rates would identify downstream consequences of the differences in responses of the conductance-based and event-based neuron models. However, we found that these metrics are too problem-specific and require additional modeling considerations, such as details on how the network is generated and connected. Controlling for these new modeling considerations would be challenging and limit the generalizability of this analysis to other neuron models. Unlike neural coding research which analyzes neuron and network models in specific mathematical and information processing contexts (e.g. [34–38]) this paper, instead, aims to assess the feasibility of a generalizable modeling framework and how learnable the output responses of a single neuron is from the limited representations of its input.

### Event-based framework as a bridge between mechanistic and phenomenological modeling

Event-based modeling is not new to network simulation; the use of INF neurons have paved the way for scaling up networks (e.g. [39]) and event communication is well integrated into NEURON [22] and other tools. What remains lacking is an efficient method to incorporate biophysical complexity at the individual neuron level for network simulations at scale. Given that the responses of conductance-based and event-based neuron models are measurably congruent, we believe that event-based modeling is a promising method for incorporating biophysical complexity at the individual neuron level in simulations at scale. Still, the specific types of error introduced by the event-based system may have unforeseen ramifications on the results of network simulations and remains a topic for further investigation [40].

The full domain of applicability of this event-based approach remains to be discovered. First, we presented here a narrow definition of the input encoding that’s limited to stimuli events and, only in some cases, the addition of prior spike events. Our event-based framework is functionally generalizable beyond the specific input encodings investigated here as it can incorporate any event of the model. For instance, incorporating the timing of the prior output spike in the encoding limited our ability to additionally consider stimuli events before it due to the specific way we set up our experiments in this study. This is an emergent limitation of the specific experiment we ran and is not the case for the framework as a whole. Second, we only used Poisson point processes to generate the timings of our stimuli and we designed the models to largely exist at a stationary balance of excitation and inhibition. The frequency of inputs is essential for characterizing the distribution of possible initial states. While we expect the random nature of our stimuli generation to account for a variety of responses, we believe our framework should be assessed for its ability to stimuli of differing kinds. Lastly, the domain of neuron models and neuroscientific concepts for which our approach is appropriate for remains to be explored. Resources like ModelDB [41] provide large numbers of published cell models that could in principle be used for such a study; unfortunately, due to the heterogeneity of neuron models and the lack of a widely convertible model interchange format, the neuron models—even the ones using NEURON—are not readily adaptable to be incorporated in such a study. That being said, exploring the specific dynamics that it works with, and those that it does not work with, is important when considering the usage of this modeling approach.

The on-event modeling platform we built in NEURON for testing the predictability of spike response from limited stimuli history could, in principle, be used to introduce machine-learned event-based approximations to the biophysical models typically used in NEURON, either for individual cells or for entire networks. Machine learning has already accelerated network simulations [42], but relative to approaches that predict state variables with machine learning, an event-based model has the potential for further speed enhancements as it can skip from synaptic event to synaptic event rather than advancing through continuous time, although the utility of this decreases as the frequency of input events increases. Efficiently generating the training set and incorporating the effects of varying biophysical parameters of interest (e.g. synaptic conductances) are major challenges, but such rapid machine-learned models offer the potential of advanced neuroscience simulation to incorporate more cells and longer run-time, bringing us closer to computationally connecting cellular dynamics to functional outcomes.
